# Property of Fluctuations of Sales Quantities by Product Category in Convenience Stores

**DOI:** 10.1371/journal.pone.0157653

**Published:** 2016-06-16

**Authors:** Gaku Fukunaga, Hideki Takayasu, Misako Takayasu

**Affiliations:** 1 Department of Computational Intelligence and Systems Science, Interdisciplinary Graduate School of Science and Engineering, Tokyo Institute of Technology, Midori-ku, Yokohama, Japan; 2 Advanced Data Analysis and Modeling Unit, Institute of Innovative Research, Tokyo Institute of Technology, Midori-ku, Yokohama, Japan; 3 Sony Computer Science Laboratories, Shinagawa-ku, Tokyo, Japan; East China University of Science and Technology, CHINA

## Abstract

The ability to ascertain the extent of product sale fluctuations for each store and locality is indispensable to inventory management. This study analyzed POS data from 158 convenience stores in Kawasaki City, Kanagawa Prefecture, Japan and found a power scaling law between the mean and standard deviation of product sales quantities for several product categories. For the statistical domains of low sales quantities, the power index was 1/2; for large sales quantities, the power index was 1, so called Taylor’s law holds. The value of sales quantities with changing power indixes differed according to product category. We derived a Poissonian compound distribution model taking into account fluctuations in customer numbers to show that the scaling law could be explained theoretically for most of items. We also examined why the scaling law did not hold in some exceptional cases.

## Introduction

In recent years with the progress and spread of information technologies and the Internet, data superior in both quality and quantity have been accumulating. Many are striving to utilize the laws and knowledge obtained from detailed analyses of these data in practical economics and to improve their daily lives. Human purchasing behavior around products and services is no exception. Numerous companies are keeping point of sales (POS) data on minute and second time-scales for all products at their stores, as well as on what and how many products are sold to each customer and where. Analyses of these data had practical and academic dimensions—such as the distribution of monetary amounts paid by customers for single-time purchases [1], product life [2], price elasticity [3]—that led to discussions of important characteristics based on quantitative observations.

The POS data accumulated over a specific time period enable the study of dynamic characteristics such as changes in the relationships between competing products, time series of sales quantities [4] and its fluctuations [5, 6]. Companies engage in advertising, price cuts, and other promotions, as well as sales on certain weekdays or days of the month, all of which have major impacts on increases and decreases in sales. These items are also being studied, and methods using models such as state space model have been proposed [7–10].

Meanwhile, Robert [5] studied the distribution of sales quantities time series and autocorrelation while excluding sales data from price reduction and sales promotion periods. These time series fluctuations are inherent in goods and services markets and are difficult for companies to predict and control. Companies require knowledge of not only the means of sales amount time series but also their distributions and fluctuations. For example, to determine the effectiveness of a sales promotion, a company needs information about statistical distributions when there is no promotion [11]; even when mean sales amounts are the same, considering inventory or opportunity loss, companies have greater difficulty deciding delivery amounts for products with large fluctuations. It is essential, especially for convenience stores and other small retailers that handle thousands of products every day, to efficiently and accurately follow all processes from production to product delivery. Production lines at factories produce many products for many different stores; they must know not only the size of sales-quantity time series fluctuations for smaller domains such as single products for single stores but also the fluctuation sizes for major domains (i.e., the totals for their customers over many stores and many products).

The proportionality relationship of a quantity associated with a variable representing scale is generally called a scaling law. Scaling law for fluctuations as a function of the mean value began with Taylor’s studies of the means and fluctuations in the numbers of individuals in natural ecology [12]; these were followed by extensions into monetary markets [13–16], networks [17, 18] and other various fields [19–22]. Sano et al. [23, 24] studied the frequencies of word appearances on Internet blogs and showed that they could be explained by combining fluctuations in an internal system factor (e.g., written quantities of individual bloggers) with those in an external factor (e.g., total blogger numbers). An internal factor for product sales quantities would be fluctuations in the number of items purchased by a single person, while an external factor would be fluctuations in customer quantities. Thus, considering these two fluctuations simultaneously should enable the construction of a fluctuation scaling law for individual products’ sales-quantities time series.

Accordingly, this paper uses POS data on convenience stores to analyze a scaling law for sales-quantities time series fluctuations when customer numbers are changed via changes in time scales (of one day or one week) and space scales (of one store or multiple stores). We also derive a fluctuation scaling model by constructing a model that simultaneously considers fluctuations due to number of products purchased by a single customer and customer-quantity fluctuations, and we compare between the scaling laws introduced by the model and actual scaling laws for each product category. Our study observes and models scaling laws concerning not only limited numbers of products within the limited parameter of products in stores but also varieties of products, including foodstuffs and non-foodstuffs, at multiple stores.

## Analysis of actual data

### Analyzed POS data

**Fig 1:**
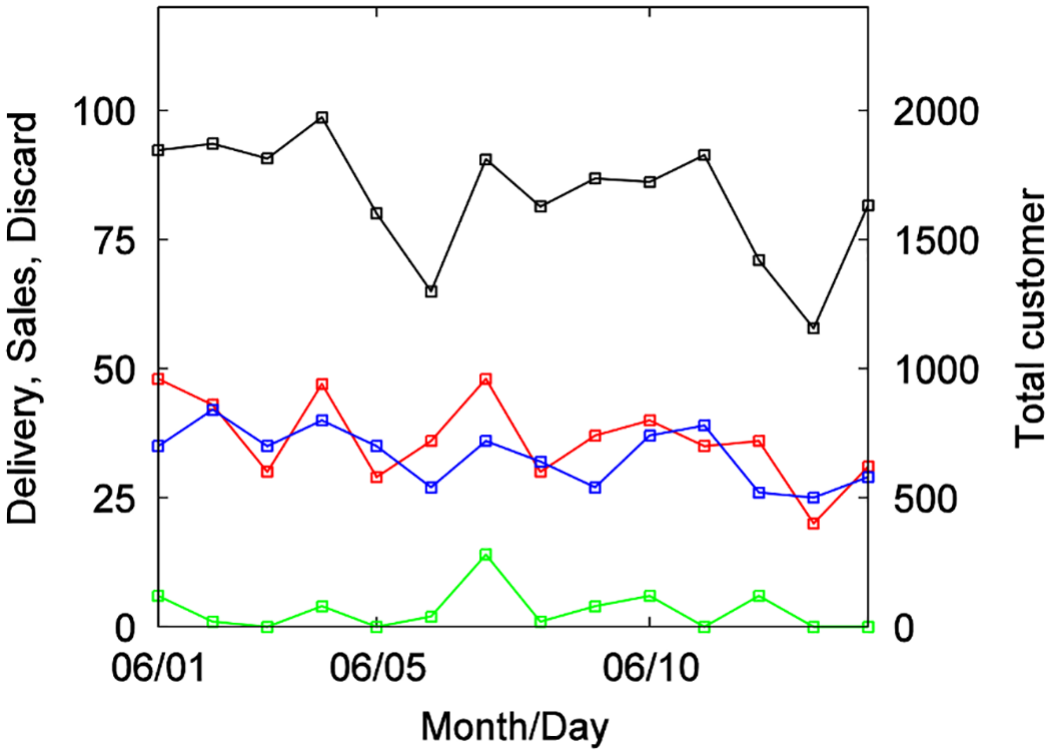
Time series of delivery quantities for a single product, rice ball at a store (red), sales quantities (blue), discard quantities (green), total customer numbers (black).

The POS data used for analysis were taken from 158 chain stores of a major convenience store company in Kawasaki City, Kanagawa Prefecture, Japan. The data cover the five month period from June 1 to October 31, 2010. These 158 stores had continuous operations during the study period (with operations commencing before June 1 and without any store closing during the period). The data were divided into two physical localities, one with 77 stores, and the other with 81. Two types of data were used for analysis: receipt data and daily data. Receipt data are recorded every time a customer completes a purchase at the cash register; these data comprise sales date, sales time, store number, store location, the sequential customer number for that day at that store, product category, product code, product name, sales quantity, and sales monetary amount. Sales time is recorded in minutes. Product categories are divisions by product type, with a total of 60 categories. When a customer submits a point card at the time of purchase, the card’s ID is recorded. However, our study computed sales quantities regardless of whether such a card was submitted. These data also include data on returned items; in such cases, the items recorded are subtracted from the total sales quantity amounts. However, as returns are extremely rare, returned items have virtually no effect on the calculations in this paper. Our second data type, daily data, indicates number of products delivered, sales quantities, and discard quantities; items recorded are date of sale, store number, store location, product category, product code, product name, delivered quantities, sales quantities, and discard quantities. Using these data types, one can recreate various time series, such as in Fig 1. This shows time series for delivery quantities, sales quantities, and discards for individual days for a single product, rice ball at a specific store, as well as a time series for total customer quantities (i.e., anyone who purchased any item) at that store for each day.

### Analyzed products

The analyzed products were the top 10 products in terms of sales quantities for all product groups (within each product category) for which at least one item was sold during one day at any one store. In some cases, there were fewer than 10 products within a single category that fulfilled these conditions. In some categories, no product fulfilled these conditions; these categories were excluded from analysis. Of all 60 categories, 45 were used for analysis (30 foodstuffs and 15 non-foodstuffs; see Table 1). For products that included any discount period, sales data within the period were excluded from the time series to exclude the effects of monetary discounts. By changing time scales (i.e., one day or one week) and space scales (i.e., one store or multiple stores) for the time series, we investigated the means and standard deviations for the sales-quantity time series for various customer numbers.

**Table 1:** Product categories and the coefficients of the variation (*σ*_*N*_/*μ*_*N*_) for each week’s totals for all stores.

Foodstuff categories				Non-foodstuff categories	
Beer	0.059	Candies1	0.080	Magazines	0.093
Japanese Sake	0.101	Candies2	0.104	Newspapers	0.041
Chuhai alcohol	0.052	Snack candies	0.085	Menswear	0.097
Rice balls	0.060	Instant noodles	0.094	Health care items	0.096
Sandwiches	0.055	Spices	0.030	Tissues	0.052
Noodles products	0.182	Rice	0.069	Shampoos and oral care products	0.056
Salads and soups	0.055	Gelled products	0.104	Detergents, cleaning	0.104
Desserts	0.047	Snack items1	0.041	Cleaning material	0.087
Fried items	0.120	Frozen foods	0.213	Hair-styling	0.194
Packaged beverages	0.072	Ice creams	0.291	Goods featuring popular character	0.155
Cheese	0.057	Soft drinks	0.098	Office supplies	0.063
Eggs	0.070	Nutrition drinks	0.137	Electrical items and umbrellas	0.098
Snack items1	0.168	Nutrition solids	0.129	Stamps	0.078
Breads1	0.051	Candies3	0.765	Coffee	0.043
Breads2	0.044	Other foodstuffs	0.166	Oversize garbage sticker	0.093

### Analysis results for sales-quantity time series for one product, one store, per one day

**Fig 2:**
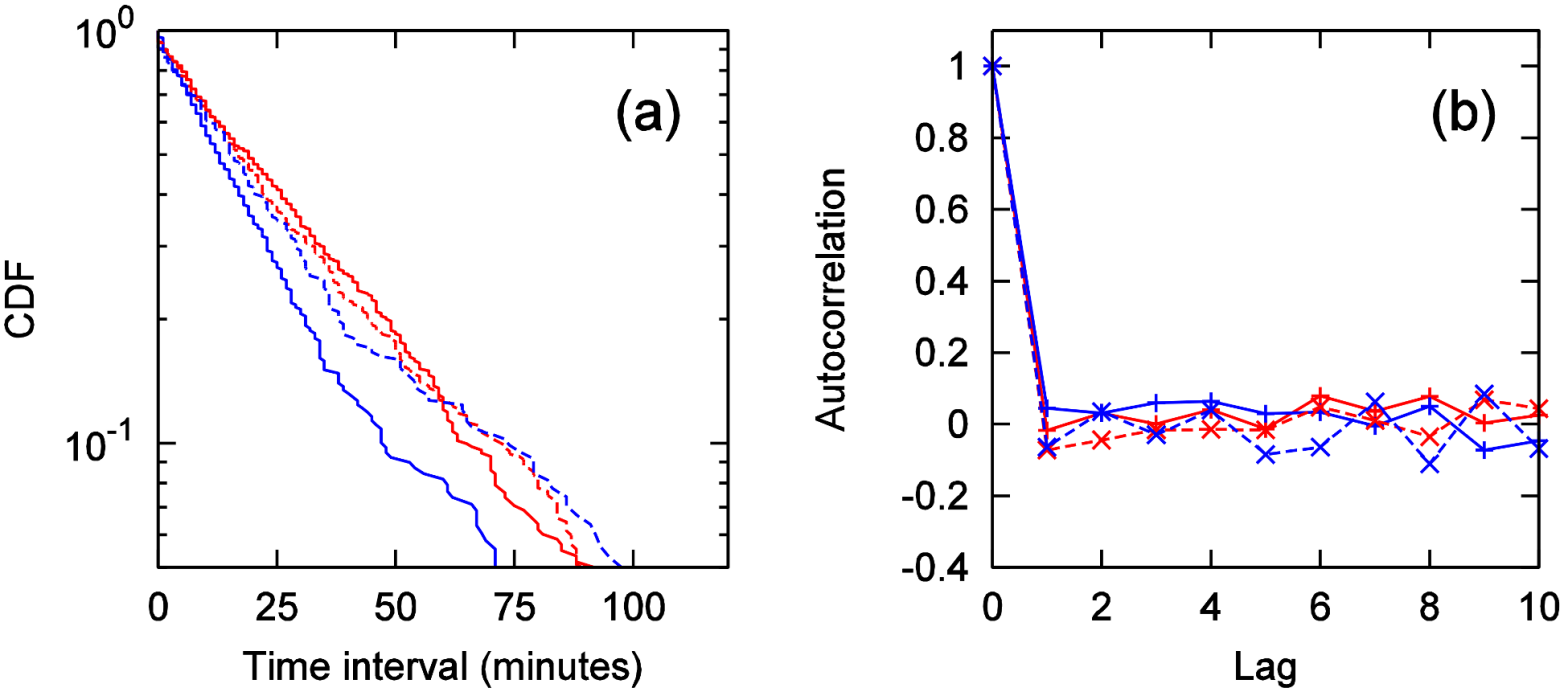
(a) Cumulative distribution of time interval (minutes) in semi-log plot: daytime on weekday (red solid line), daytime on non-weekday (blue solid line), evening on weekday (red dashed line), evening on non-weekday (blue dashed line). (b) Autocorrelation of time interval.

First, we analyze the smallest scale, sales-quantity time series for one product, one store, per one day. Fig 2(a) shows the semi-log plot of cumulative distribution of sales time intervals for two time frames—weekdays and non-weekdays (e.g., weekends, holidays)—for one product, soft drinks, at a single store. In this paper, a time interval is the time from the sale of one product until the next time an identical product is sold again. For sales-quantity time series, the periodicity of weekdays is usually considered; thus, our analysis divided the days of the week into “weekdays” and “non-weekdays”. In our results, the “daytime” timeframe includes data from 11:00 a.m. to 2:59 p.m., and the “evening” timeframe includes data from 6 p.m. to 9:59 p.m. The distributions are displayed by individual (approximately) straight lines, showing that sales-quantity time intervals can be approximated with exponential distributions with differing statistical means.

Fig 2(b) shows the autocorrelation of the time series of sales-quantity time intervals. (CDFs and autocorrelations of some other categories are shown in S1 Fig.) The result for each is near 0. The Ljung-Box test [25] was performed for these. Under the null hypothesis that the autocorrelation coefficient is 0, the test statistic *Q* as defined in Eq (1) follows the chi-square distribution of degree *n*:

$$\begin{array}{c} Q=T\left(T+2\right)\sum_{k=1}^{n}\frac{{\hat{\rho_{k}}}^{2}}{T-k} \end{array}$$ (1)

Here, *T* and *ρ*_*k*_ are the number of sample and autocorrelations of time series data quantity with lag *k*, respectively. In the results of tests that changed *n* to 1 through 10, there was no rejection of the null hypothesis at a 5% significance level (see S1 Table).

For this store, the mean *μ* and standard deviation *σ* of the product’s sales quantity time series for every Friday are 40.9 and 7.0, respectively, showing the following relationship:

$$\begin{array}{c} \sigma \approx \mu^{\frac{1}{2}} \end{array}$$ (2)

Some studies (e.g., [26]) assume that sales quantities for single products (i.e., one item) follow a Poisson process; for sales of one item at one convenience store per one day, this assumption is reasonable.

### Analysis results for a variety of scales

By changing the scale of customer quantities observing with different time and space scales, we investigated the relationship between the means and standard deviations of sales and customer quantities.

#### Sales-quantity time series

**Fig 3:**
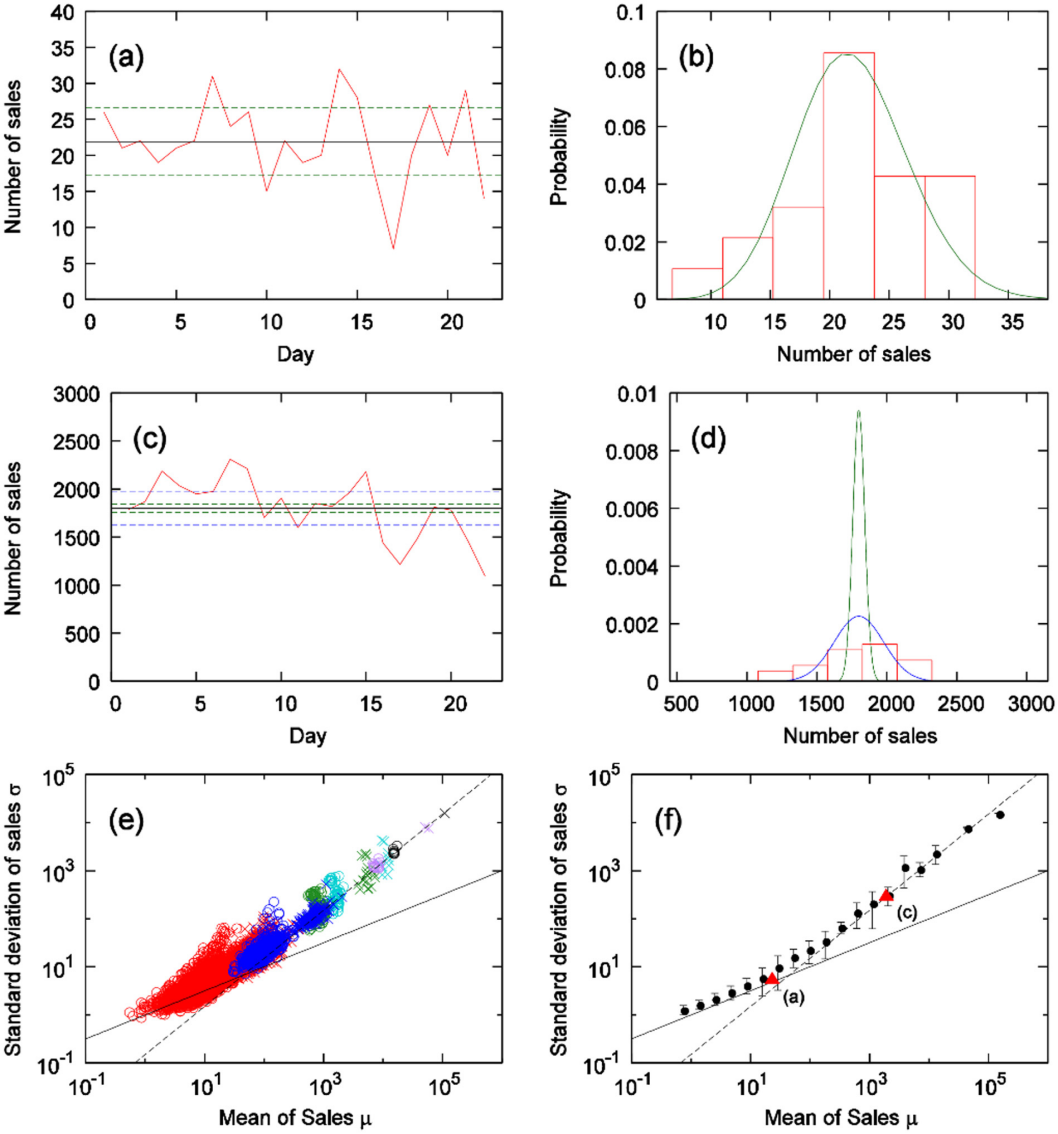
**(a),(b) Sales-quantity time series and distribution of 1product, 1store, each day of the week. (c),(d) Sales-quantity time series and distribution of 1product, all stores within the city, each day of the week**. In (b),(d), green line shows a Poisson distribution whose mean is the same as the mean of real data. In (d), blue line is a Gaussian distribution whose mean is the same as the mean of real data and the standard deviation is 0.1 times of the mean of real data. In (a),(c), green and blue dashed lines represent the standard deviations of (b),(d), respectively, and black line is the mean of real data. **(e) Relationship between mean and standard deviation of sales quantities in the soft drinks category**. Regarding plot points, for each day of week: circle, weekly totals: cross, 1 product on 1 store: red, all products on 1 store: blue, 1 product within locality: green, all products within locality: magenta, 1 product within city: cyan, all products within city; black. Black solid line shows $\sigma =\sqrt{\mu }$, and black dashed line shows *σ* = 0.15*μ*. **(f) Means of (e) with error bars given by the standard deviation**. Two red triangles indicate mean and standard deviation of (a) and (c).

For each product category, the following 12 combinations of sales-quantity time series were analyzed:

1. 1product, 1store, each day of the week (See Fig 3(a) and 3(b))
2. 1product, 1store, weekly totals
3. 1product, all stores within a locality, each day of the week
4. 1product, all stores within a locality, weekly totals
5. 1product, all stores within the city, each day of the week (See Fig 3(c) and 3(d))
6. 1product, all stores within the city, weekly totals
7. all 10 products, 1store, each day of the week
8. all 10 products, 1store, weekly totals
9. all 10 products, all stores within a locality, each day of the week
10. all 10 products, all stores within a locality, weekly totals
11. all 10 products, all stores within the city, each day of the week
12. all 10 products, all stores within the city, weekly totals

Fig 3(e) and 3(f) shows the means *μ* and standard deviations *σ* of product sales-quantity time series for the soft drinks category. We see that, when *μ* is in a region sufficiently smaller than 10, Eq (2) holds; when *μ* is in a region sufficiently larger than 10, this relationship does not hold; rather, the following holds:

$$\begin{array}{c} \sigma \propto \mu \end{array}$$ (3)

This type of transition of power law is called Taylor’s law, and in our study, the region (statistical domain) where Eq (2) holds is called the “Poisson area”, and the region where Eq (3) holds is called the “non-Poisson area”.

**Fig 4:**
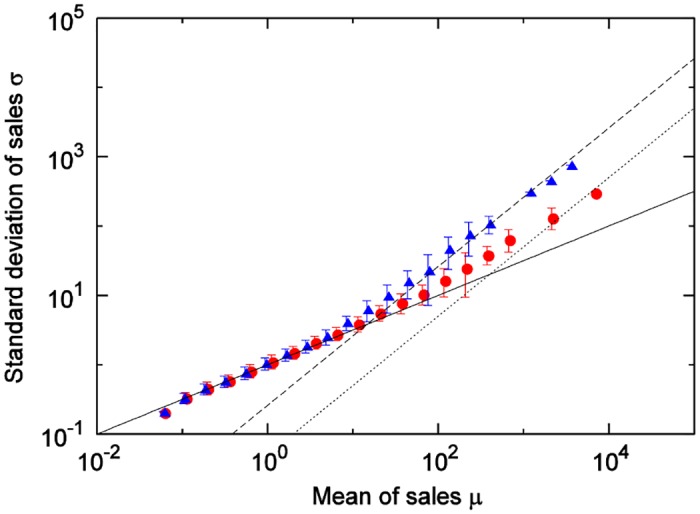
Relationship between mean and standard deviation of sales quantities by category: cheese (red) and hair-styling (blue). Solid, dashed and dotted line show $\sigma =\sqrt{\mu }$, *σ* = 0.26*μ*, and *σ* = 0.05*μ* respectively.

We next investigate the differences according to product category. Fig 4 shows the results of a comparison between the cheese category and the hair-styling products category. When the sales quantity means of both categories are in the small region, Eq (2) holds; when both are in the large region, Eq (3) holds. However, the boundary values where the movements of the means and the standard deviations change from Eqs (2) and (3) differ.

#### customer-quantity time series

Before presenting the results of the customer-quantity time series analysis, we first comment on the analysis method for customer quantities used in this paper. In this paper, individual customers are not distinguished; rather, the numbers of purchases completed at the register are denoted as “customer quantities”. For example, suppose the same person makes three purchases in one day at the same convenience store. If individuals were distinguished, this person would be counted as one customer; since individuals cannot be distinguished in POS data, however, this person would be counted as three persons in our study. Customers are also analyzed by classifying these for each product category. If a customer were to purchase any product within a category targeted for analysis, regardless of the number of products bought, this would be counted as one person. Conversely, even if a customer were to purchase several items of products not within a targeted category, they would not be counted within the customer quantities for the targeted categories. If a customer were to purchase two items from different targeted categories, more customers would be counted than the actual customer quantities (number of purchases completed at the register) since these would be counted as individual purchases within each category. For each product category, the following six combinations of customer-quantity time series were analyzed:

1. 1store, each day of the week
2. 1store, weekly totals
3. all stores within a locality, each day of the week
4. all stores within a locality, weekly totals
5. all stores within the city, each day of the week
6. all stores within the city, weekly totals

**Fig 5:**
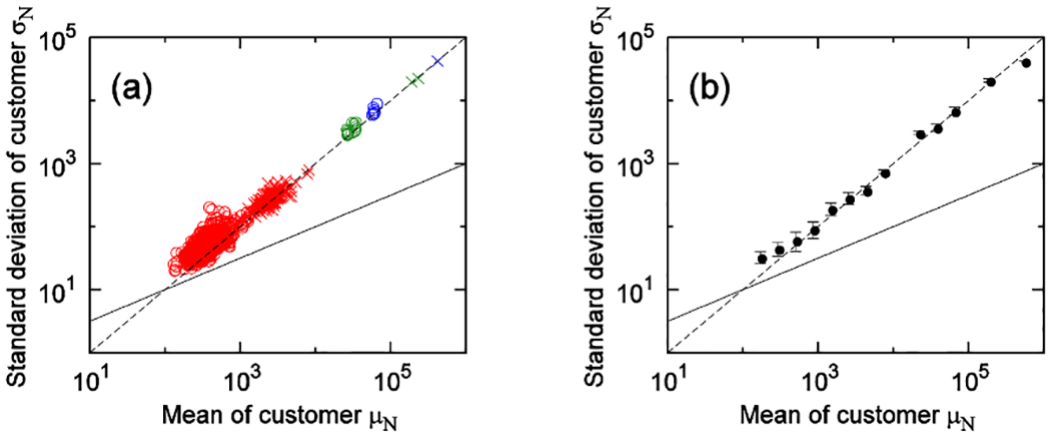
**(a) Relationship between mean and standard deviation of customer-quantities time series for the soft drink category**. Regarding plot points, for each day of week: circle, weekly totals: cross, on 1 store: red, within 1 locality: green, within city: blue. Black solid line shows $\sigma_{N}=\sqrt{\mu_{N}}$, and black dashed line shows *σ*_*N*_ = 0.10*μ*_*N*_. **(b) Mean of (a)**.

Fig 5(a) and 5(b) shows the results of the customer-quantity time series for the soft drinks category. Unlike in the results for sales-quantity time series, Eq (3) holds in all regions (statistical domains).

**Fig 6:**
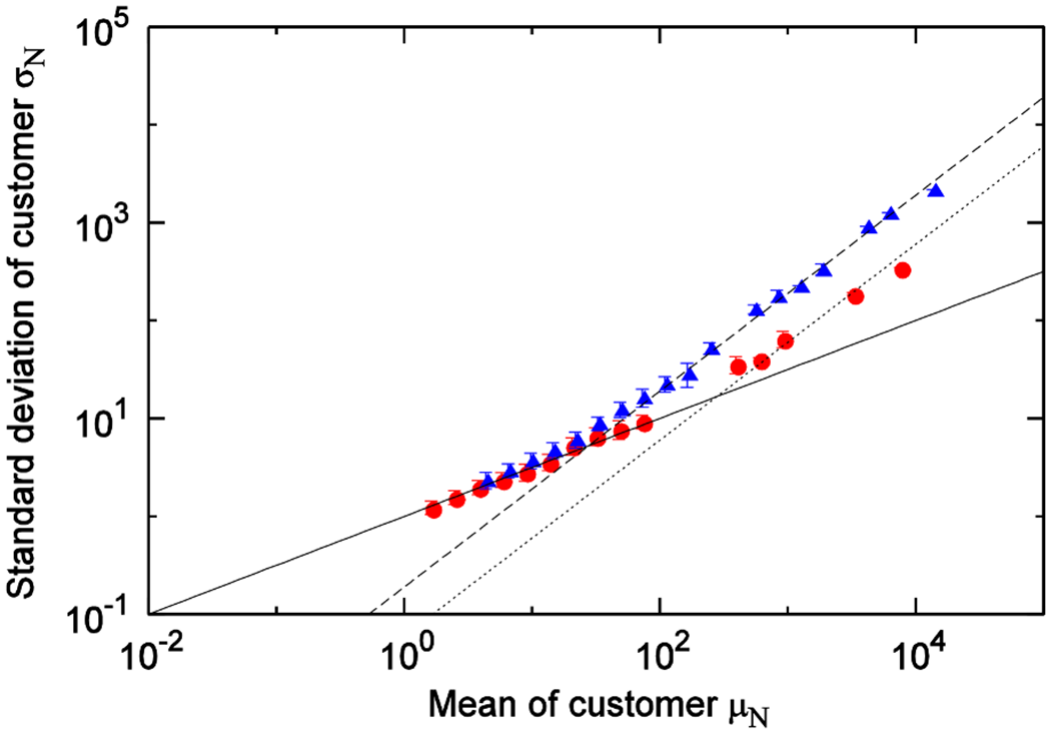
Relationship between mean and standard deviation of customer-numbers time series by category: cheese (red) and hair-styling (blue). Solid, dashed and dotted line show $\sigma_{N}=\sqrt{\mu_{N}}$, *σ*_*N*_ = 0.19*μ*_*N*_, and *σ*_*N*_ = 0.06*μ*_*N*_ respectively.

We next investigate differences according to product category. Fig 6 shows the results of a comparison between the cheese category and the hair-styling products category. As with the results for the sales-quantity time series, when the customer quantity means of both categories are in the small region, Eq (2) holds; when both are in the large region, Eq (3) holds. Moreover, the boundary value points where the movements of the means and the standard deviations change are different. As Fig 5 shows, the customer-quantity time series for one store, one product, and one day—the smallest scale—fell within the non-Poisson area for the soft drinks category because it is larger in terms of customer scale than of the cheese or hair-styling products categories, which are the same scale. If one were to observe the soft drinks category at an even smaller time scale (for example, every hour), it would likely fall within the Poisson area.

### Summary of analysis results

The results of the analysis of actual data are summarized below:

1. For the statistical mean *μ* and standard deviation *σ* of the sales-quantity time series, when *μ* ≪ *A*, then *σ* ≈ *μ*^1/2^, and when *μ* ≫ *A*, then *σ* ∝ *μ*. Here, A is a constant determined by the product category.
2. For customer-quantity time series classified by category, the above holds.
3. In the Poisson area, the sales process can be approximated by the Poisson process.
4. Even though the scaling of customer quantities is non-Poisson-like (*σ* ∝ *μ*) as shown in Fig 5, the scaling of sales quantities can be Poisson-like ($\sigma =\sqrt{\mu }$) from result of soft drink category as shown in Fig 3(f) in the range *μ* < 10^1^.

Hereafter, compound distribution will be constructed, and the reproducibility of the characteristics described above will be discussed.

## Derivation of scaling laws for fluctuations using compound distributions

In this section, we derive scaling laws for fluctuations in sales quantities by using a model that simultaneously considers fluctuations in the number of items purchased at one time and fluctuations in customer quantities (number of purchases completed at the register). The next section compares the results of this section with the actual data.

Here, *X*_*i*_ is a random variable showing the number of items of a single product purchased at a single shopping event by customer *i*. *N* is the customer quantity (number of purchases completed at the register) of the relevant product category. We then consider that *X* summed *N* times equals the total sales quantity *S* of one product. That is,

$$\begin{array}{c} S=X_{1}+X_{2}+\cdots +X_{i}+\cdots +X_{N-1}+X_{N} \end{array}$$ (4)

Here, each *X*_*i*_, (*i* = 1, 2, ⋯, *N* − 1, *N*) independently follows the same distribution and becomes a series of random variables with no time (temporal) or space (spatial) dependency. The characteristics of customers are considered to be identical and not dependent on the day of the week or the store. Customer quantity *N* is also considered a random variable not dependent on time. In general, the distribution followed by *S* as defined in Eq (4) is called a “compound distribution” wherein the distribution followed by *X* is compounded with the distribution followed by *N* (for compound distribution, see [27]). The probability density functions of *X*, *N*, and *S* are, respectively, *f*_*X*_, *f*_*N*_, and *f*_*S*_, and the expected values for each of these are *E*_*X*_, *E*_*N*_, and *E*_*S*_. The expected values of arbitrary function *g* can thus be shown, respectively, as

$$\begin{array}{c} E_{X}\left[g\left(X\right)\right]=\sum_{x}g\left(x\right)f_{X}\left(x\right) \end{array}$$ (5)

$$\begin{array}{c} E_{N}\left[g\left(N\right)\right]=\sum_{n}g\left(n\right)f_{N}\left(n\right) \end{array}$$ (6)

$$\begin{array}{c} E_{S}\left[g\left(S\right)\right]=\sum_{s}g\left(s\right)f_{S}\left(s\right) \end{array}$$ (7)

Using these, the respective means *μ* and standard deviations *σ* of *X*, *N*, and *S* are defined as follows:

$$\begin{array}{cccccc} \mu_{X} & = & E_{X}\left[X\right] & \;\;\sigma_{X} & = & \sqrt{E_{X}\left[X^{2}\right]-E_{X}{\left[X\right]}^{2}}\\ \mu_{N} & = & E_{N}\left[N\right] & \;\;\sigma_{N} & = & \sqrt{E_{N}\left[N^{2}\right]-E_{N}{\left[N\right]}^{2}}\\ \mu_{S} & = & E_{S}\left[S\right] & \;\;\sigma_{S} & = & \sqrt{E_{S}\left[S^{2}\right]-E_{S}{\left[S\right]}^{2}} \end{array}$$

The scaling relationship of the mean *μ*_*S*_ and the standard deviation *σ*_*S*_ of sales quantity *S* can thus be strictly derived (see S1 Appendix):

$$\begin{array}{c} \sigma_{S}=\sqrt{\frac{\sigma_{X}^{2}}{\mu_{X}}\mu_{S}+\text{CV}{\left(N\right)}^{2}\mu_{S}^{2}} \end{array}$$ (8)

Here, *CV*(*N*) expresses the coefficient of the variation in customer quantity and is *CV*(*N*) = *σ*_*N*_/*μ*_*N*_. From our hypothesis, the square root $\sigma_{X}^{2}/\mu_{X}$ of Eq (8) is not dependent on time or space scales, and *CV*(*N*)^2^, from the actual data, is 1/*μ*_*N*_ in the Poisson area, and, in the non-Poisson area, a constant whose value differs for each product category. Thus, from Eq (8),

$$\begin{array}{cc} \sigma_{S}=\sqrt{\frac{\sigma_{X}^{2}}{\mu_{X}}+\mu_{X}}\cdot \mu_{S}^{1/2} & \;(\mu_{S}\ll A_{m}) \end{array}$$ (9)

$$\begin{array}{cc} \sigma_{S}\approx \text{CV}\left(N\right)\cdot \mu_{S} & \;(\mu_{S}\gg A_{m}) \end{array}$$ (10)

are derived. *A*_*m*_ is the crossover value where the scaling law changes, and is

$$\begin{array}{c} A_{m}=\frac{\sigma_{X}^{2}}{\mu_{X}}\text{CV}{\left(N\right)}^{-2} \end{array}$$ (11)

Thus, by considering the fluctuations in numbers of items purchased and in customer quantities for each time point, one can express the difference in the scaling laws for sales quantity fluctuations in the small and large (statistical) regions.

The scaling law in the region where *μ*_*S*_ ≫ *A*_*m*_ is satisfied does not contradict the scaling law in the Poisson area observed in the actual data, as shown in the previous section. Conversely, in the region where *μ*_*S*_ ≪ *A*_*m*_ is satisfied, although *σ*_*S*_ is proportional to $\mu_{S}^{1/2}$, the proportionality coefficient is dependent on the distribution of *X*. Moreover, when one assumes that, as with the soft drinks category, the scaling of customer quantities is non-Poisson-like in all regions, in the scaling law for the region where *μ*_*S*_ ≪ *A*_*m*_ is satisfied, one can ignore the second term of the square root of Eq (8); this thus becomes $\sigma_{S}\approx \sqrt{\sigma_{X}^{2}/\mu_{X}}\cdot \mu_{S}^{1/2}$. Likewise, in this case, although *σ*_*S*_ is proportional to $\mu_{S}^{1/2}$, the proportionality coefficient is dependent on the distribution of *X*.

The statistical models for the number of items purchased by a single individual are the negative binomial distribution (NBD) [28–30] model, which postulates that Poisson distribution whose mean follows gamma distribution, and the beta-binomial distribution (BBD) [31–33] model, which postulates that binomial distribution whose purchase probability follows beta distribution (considerations regarding the application of the NBD and BBD models for the purchase of *X* number of items at one time are provided S2 and S3 Appendix.) One must note, however, that these models are not concerned with the number of items purchased at one time but rather the number of items purchased within a specific time period. More simply than in these models, this paper assumes that the purchase of *X* number of items at one time is a simple Poisson distribution of the mean *λ*. Thus, from $\mu_{X}=\sigma_{X}^{2}=\lambda$, the scaling law Eq (8) becomes

$$\begin{array}{c} \sigma_{S}=\sqrt{\mu_{S}+\text{CV}{\left(N\right)}^{2}\mu_{S}^{2}} \end{array}$$ (12)

and Eqs (9) and (10) become

$$\begin{array}{c} \sigma_{S}=\sqrt{1+\mu_{X}}\cdot \mu_{S}^{1/2}\;\;\;\;\left(\mu_{S}\ll A_{m}=\text{CV}{\left(N\right)}^{-2}\right) \end{array}$$ (13)

$$\begin{array}{c} \sigma_{S}\approx \text{CV}\left(N\right)\cdot \mu_{S}\;\;\;\;\left(\mu_{S}\gg A_{m}=\text{CV}{\left(N\right)}^{-2}\right) \end{array}$$ (14)

Furthermore, when it is assumed that *μ*_*X*_ ≪ 1 (for example, for the product with the highest sales in the soft drink category, *μ*_*X*_ = 0.030.), then, when *μ*_*S*_ ≪ *A*_*m*_, this becomes $\sigma_{S}\approx \mu_{S}^{1/2}$. The same equation holds when the scaling of customer quantities is non-Poisson-like in all ranges. The scaling of customer quantities is then in both Poisson and non-Poisson areas, and the scaling of sales quantities is expressed as

$$\begin{array}{c} \sigma_{S}\approx \sqrt{\mu_{S}+\text{CV}{\left(N\right)}^{2}\mu_{S}^{2}} \end{array}$$ (15)

and the boundary value *A*_*m*_ where there is a change from $\sigma_{S}\propto \mu_{X}^{1/2}$ to *σ*_*S*_ ∝ *μ*_*X*_ becomes

$$\begin{array}{c} A_{m}=\text{CV}{\left(N\right)}^{-2} \end{array}$$ (16)

Here, *CV*(*N*) is the coefficient of the variation in customer quantities within the non-Poisson area. Note that, when it is postulated that *X* follows a binomial distribution (using the BBD model), the same results can be derived (see S3 Appendix). A noteworthy characteristic here is that, since, with Eq (15), parameters due to *X* do not appear, a scaling law can be shown only for the coefficient of variation (a constant) of customer quantities. The next section compares the scaling laws from the POS data to the scaling law of Eq (15).

## Comparison of actual data to the theory

**Fig 7:**
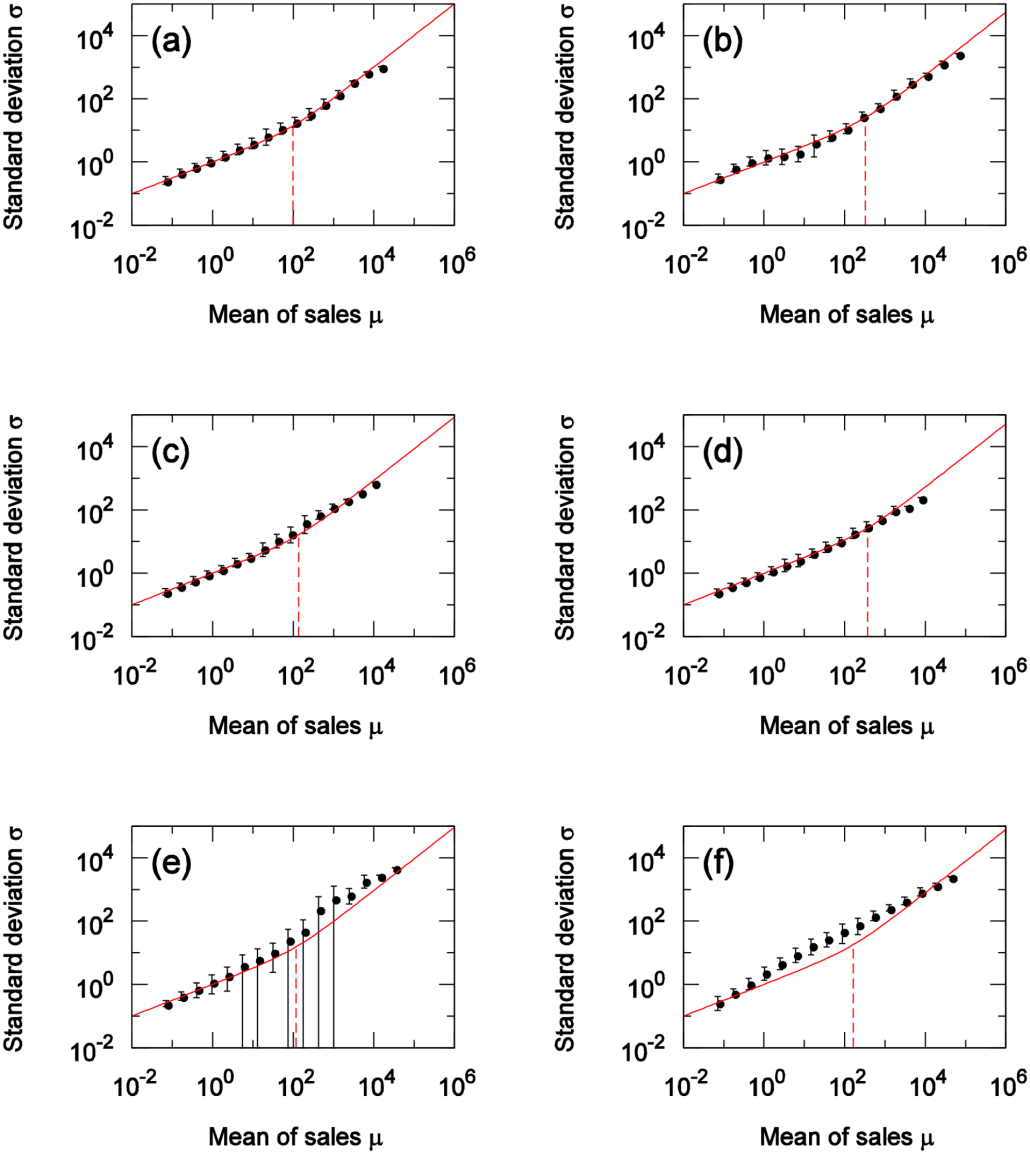
Comparison of actual data and theory. (a) Japanese sake (rice wine), (b) Soft drinks, (c) Eggs, (d) Tissues, (e) Magazines, (f) Stamps. Solid and dashed line show Eq (15), the crossover value *A*_*m*_ respectively.

**Fig 8:**
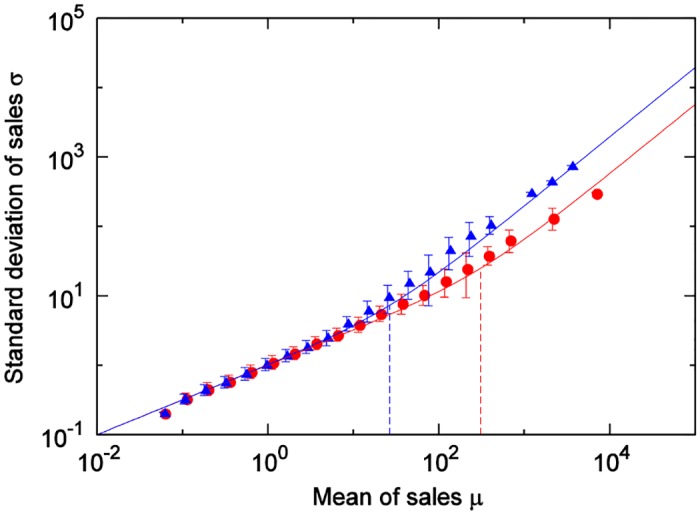
Comparison of reproducibility for two item categories: cheese (red) and hair-styling (blue). Two solid line show Eq (15) and dashed lines indicate crossover value *A*_*m*_ respectively. Plots are same as in Fig 4.

Here, we compare Eq (15), where the number of items *X* purchased at one time follows a Poisson distribution, with the results of the scaling laws derived from actual data. For each category, we calculate the coefficient of the variation in customer-quantity time series for weekly totals at all stores in city (actual measured values for each category are found Table 1), and use the results as CV(N) in Eq (15). Fig 7 shows the comparison results for 6 of the 45 categories. In each of these figures, Solid lines show Eq (15) and dashed lines show the boundary value *A*_*m*_ as calculated by Eq (16). From Fig 7, it is clear that, for many categories, scaling laws in the Poisson and non-Poisson areas could be expressed with Eq (15). Fig 8 shows the results for the cheese category and the hair-styling products category. Fig 8 also shows how, with the categorization of customers, the use of the coefficients of the variation in these categories enables the expression of the differences due to these categories. The stamp and magazine categories show relatively large discrepancies between the results of the actual data and those of Eq (15). Considerations are presented for these two items below, category by category.

### Regarding the stamps category

**Fig 9:**
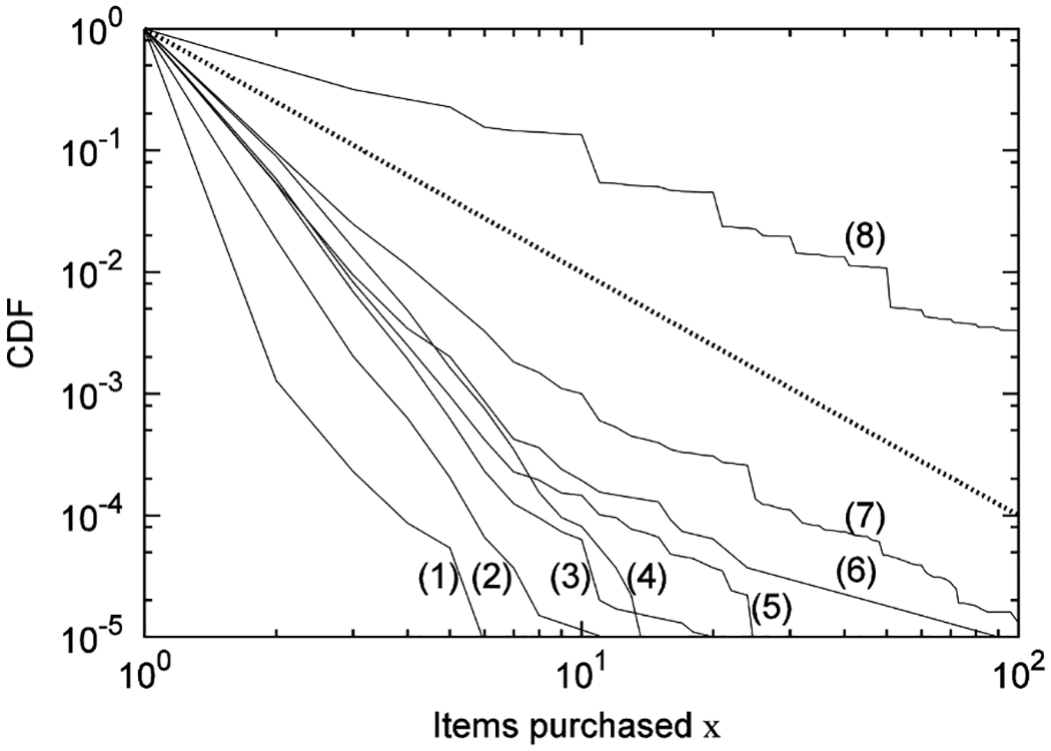
Cumulative distribution of quantities by a purchase. **(1)Magazines, (2)Eggs, (3)Salads and soup, (4)Gelled items, (5)Instant noodles, (6)Tissues, (7)Soft drinks, (8)Stamps**. Dashed line shows *x*^−2^

From Eq (9), on the Poisson area, $\sigma_{S}\propto \mu_{S}^{1/2}$ holds. However, as Fig 7(f) shows, within the Poisson area, $\sigma_{S}\propto \mu_{S}^{1/2}$ does not hold. One possible reason is that the distribution of *X* follows a power-law distribution, whose variance cannot be defined, and thus $\sigma_{S}\propto \mu_{S}^{1/2}$ did not hold. Fig 9 shows the cumulative distribution of *X* from actual data for customers who purchased one or more products, namely Pr(*X* ≥ *x*|*X* ≥ 1) for each category. When the power index *α* is less than 2 within the power-law distribution *f*(*x*) = *αβ*^*α*^/*x*^*α*+1^, (*β* ≤ *x*), variance cannot be defined (it diverges). If we assume that *X* follows a power-law distribution, Fig 9 suggests that the power index for the stamps category was less than 2, while the power index for other categories larger than 2. For a given finite dataset, a power-law distribution, with a low power index *α* gives a high mean value. This suggests that the stamps category involves an item that is easy to buy in bulk (i.e., many at one time); and this is the reason of its discrepancy with Eq (15). However, even if the mean of *X* is large, if the mean and the variance are definable, the discrepancy with the theory is not so great—at the very least, from the consideration that $\sigma_{S}\propto \mu_{S}^{1/2}$ holds in that case.

### Regarding the magazines category

**Fig 10:**
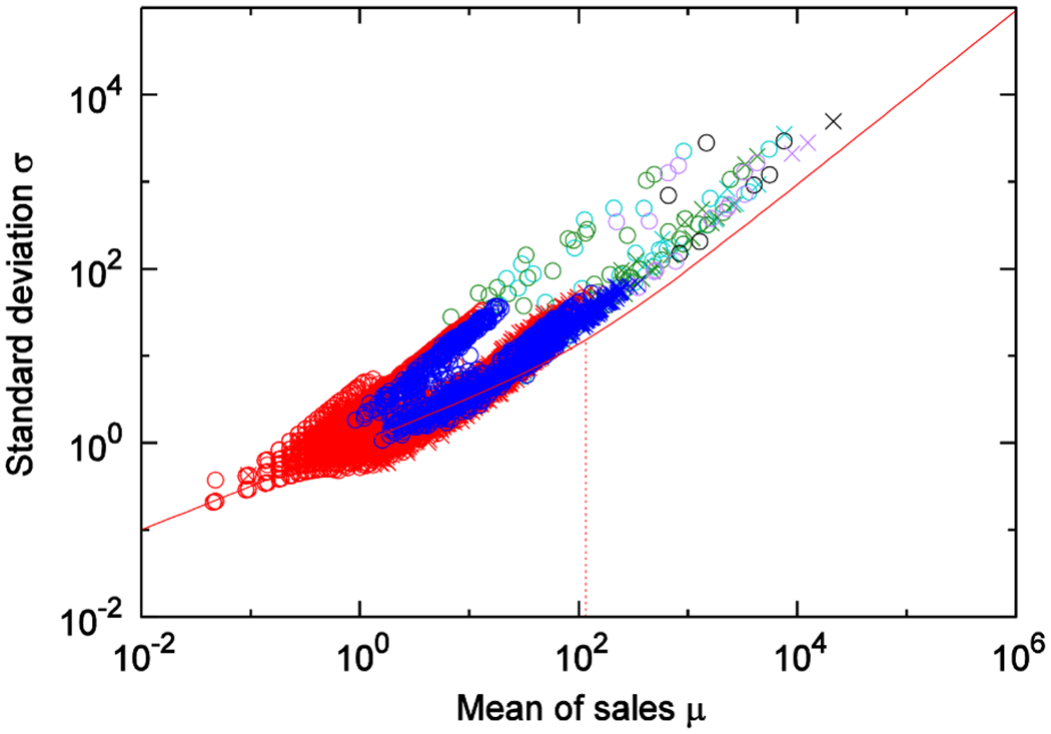
Scaling law in the magazines category. Solid and dashed lines show Eq (15), crossover value *A*_*m*_ respectively.

**Fig 11:**
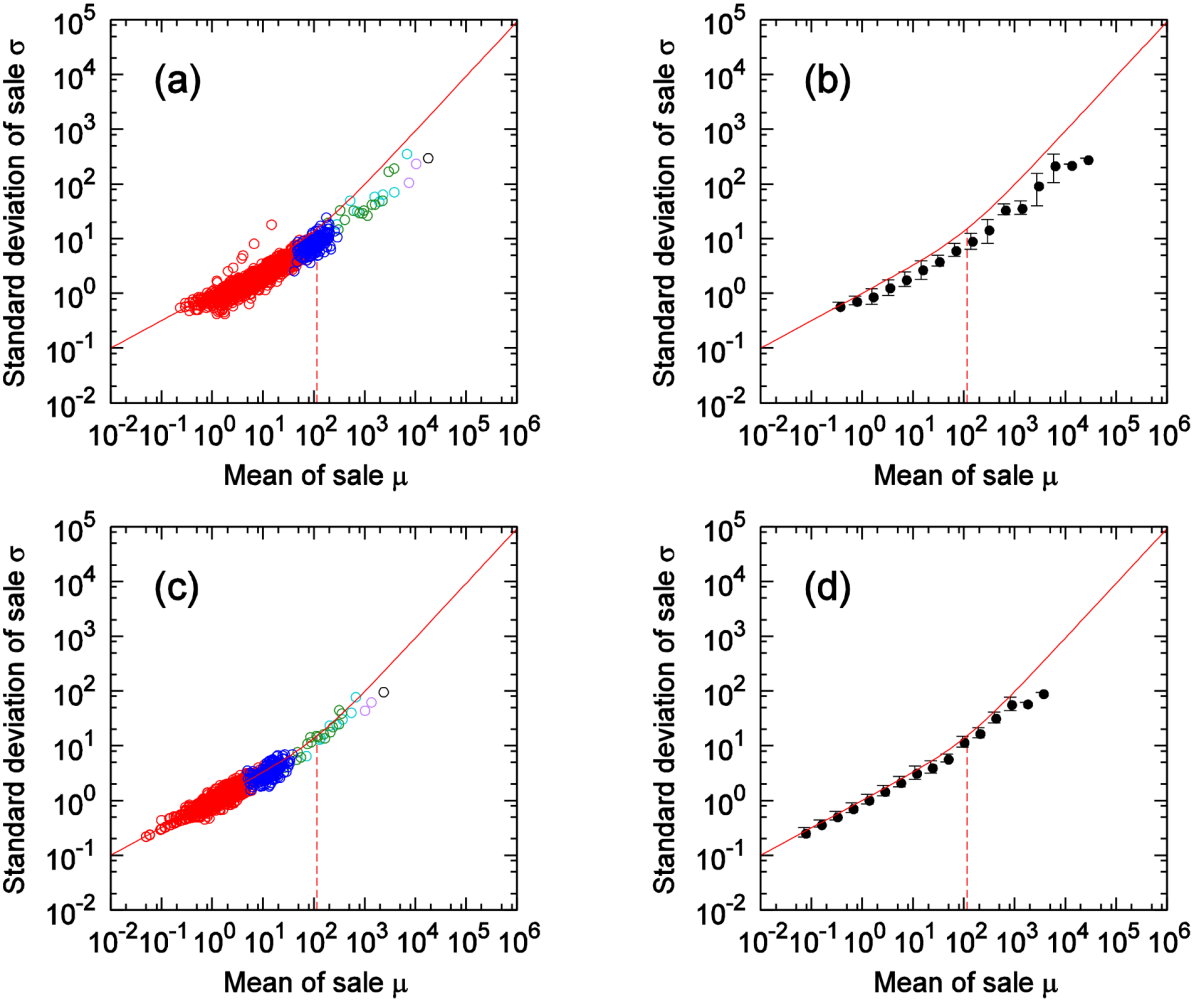
Scaling laws in the magazines category considering the date of sale of each magazine. **(a),(b) Results on date of sale. (c),(d) Results on a day after the date of sale**. Solid and dashed line show Eq (15), crossover value *A*_*m*_ respectively. (b) is the mean of (a), (d) is the mean of (c).

Fig 10 shows scatter plots of the scaling laws from the POS data for the magazine category. The actual data have wider fluctuations than do those of the theory, and the scattering of each plot point of the actual data is larger than for other categories. In Fig 9, unlike the case with the stamps category, for magazines, the mean of *X* is small, and, even under the assumption that *X* is a power-law distribution, its index is still greater than 2. Thus, the divergence between the actual data and Eq (15) is not due to the effects of bulk purchases. In the magazines category, the divergence occurs because all of the nine products targeted for analysis are weekly magazines. In general, the greatest sales of weekly magazines occur on the date of sale, and this quantity diminishes thereafter. Moreover, when the date of sale is a holiday, the magazine will often be sold on another day. For example, if the date of sale is every Monday, when the Monday is a holiday, the magazine may be sold on the immediately preceding Saturday. Studies have considered the periodicity of the days of the week. Analyses of sales quantities per day have examined for each day of the week. If the date of sale is a holiday, however, the periodicity is interrupted, making it necessary to consider periodicity as starting with the date of sale, followed by the next, and the next, and so on. Analysis results of a scaling law for date of sale are shown in Fig 11(a) and 11(b), while Fig 11(c) and 11(d) show the results of a scaling law for the day after the date of sale. As the date of sale was considered as the periodicity standard, overall scattering is reduced, and the divergence with Eq (15) becomes smaller.

Standard deviation *σ* on a Poisson area for date of sale is smaller than that derived from Eq (15), perhaps because of the existence of fixed customers: that some customers always purchase a certain magazine every week may have made this fluctuation smaller than for other categories. Danaher [32] has researched the existence of fixed customers (i.e., periodical fixed purchasers) using monthly magazine purchase data. Danaher [32] modified the BBD model described in S3 Appendix to consider fixed customers, showing that such considerations make for a better fit with actual data. The modified BBD model is called the “MBBD model”; here, with *n* indicating the number of times a magazine is published, the probability density function *f*(*X*) of the random variable *X* showing purchasing quantities within n times is expressed as

f(X)=(1-ω)(nX)B(p+X,q+n-X)B(p,q)+ωI{X=n},X=0,1,2,⋯,n (17)

*I*_{*X* = *n*}_ is an indicator function defined as, when *X* = *n*, then *I*_{*X* = *n*}_ = 1, when *X* ≠ *n*, then *I*_{*X* = *n*}_ = 0. *ω*(0 ≤ *ω* ≤ 1) shows the percentage of fixed customers (those who with probability 1 will buy a magazine); when *ω* = 0, then this reverts to the BBD model where there is no consideration of fixed customers. Here, B(*p*, *q*) is a beta function with positive parameters, *p* and *q*. The mean of *X* and the variance $\sigma_{X}^{2}$ respectively become [32]

$$\begin{array}{c} \mu_{X}=n\frac{p+\omega q}{p+q} \end{array}$$ (18)

$$\begin{array}{c} \sigma_{X}^{2}=\left(1-\omega \right)\frac{npq\left(p+q+n\right)+n^{2}\omega q^{2}\left(p+q+1\right)}{{\left(p+q\right)}^{2}\left(p+q+1\right)} \end{array}$$ (19)

To compare this with the scaling law for the date of sale of weekly magazines, *n* = 1, and the mean *μ*_*X*_ becomes

$$\begin{array}{c} \mu_{X}=\frac{p+\omega q}{p+q} \end{array}$$ (20)

When this mean is used, the variance becomes

$$\begin{array}{ccc} \sigma_{X}^{2} & = & \left(1-\omega \right)\frac{pq\left(p+q+1\right)+\omega q^{2}\left(p+q+1\right)}{{\left(p+q\right)}^{2}\left(p+q+1\right)}\\ & = & \left(1-\omega \right)q\frac{p+\omega q}{{\left(p+q\right)}^{2}}\\ & = & \left(1-\omega \right)\frac{q}{p+q}\mu_{X} \end{array}$$

hence,

$$\begin{array}{c} \frac{\sigma_{X}^{2}}{\mu_{X}}=\left(1-\omega \right)\frac{q}{p+q} \end{array}$$ (21)

When this is inserted into Eq (9), a scaling law considered fixed customers within the Poisson area of sales quantities can be derived:

$$\begin{array}{c} \sigma_{S}=\sqrt{1-\omega \frac{q}{p+q}}\cdot \mu_{S}^{1/2} \end{array}$$ (22)

Thus, since 0 ≤ *ωq*(*p*+*q*)^−1^ ≤ 1, by considering fixed customers, the MBBD model can show that, even if the means within the Poisson area is the same, the standard deviation becomes smaller than $\mu_{S}^{1/2}$. Therefore, the analysis results of this paper confirm the existence of fixed customers (subscribers) of weekly magazines at convenience stores from the scaling law of fluctuation.

## Conclusion

By analyzing POS data from convenience stores, this paper showed that Taylor’s law holds between the means and standard deviations of most of actual quantities and constructed a model to fit with the data. The constructed model enabled the simultaneous consideration of fluctuations in individual item purchase quantities and customer quantities, allowing us to reproduce the characteristics of actual scaling laws such that, for sales quantities in areas (statistical domains) with low sales quantities, the power index was 1/2, and, in areas with large sales quantities, the power index was 1. The fact that the boundary value where the power index was changed would differ for different product categories was reflected by classifying customers into categories.

We also separately analyzed the magazines and stamps categories, which have special characteristics in terms of their scaling laws. For magazines, considering the standard periodicity of weekly magazines’ dates of sale, the scaling law was shown to hold, and we also confirmed the existence of fixed customers from the scaling law, as described by Danaher [32].

The stamps category was a clear example of violation of Taylor’s law. Since the number of items purchased each time follows a power-law distribution and since the power index is less than 2, variance cannot be well defined. We showed from the distribution of actual item purchase quantities that this category showed behavior different from that of the scaling laws derived from our model.

Summarizing the results we can say that the reason why the number of sales of some categories are Non-Poisson is classified in two cases. One is the case when fluctuation of the number of customer is bigger than fluctuation of sales quantities by a purchase. The other is the case when distribution of quantities by a purchase follows a power-law distribution whose variance cannot be well-defined (it tends to diverge for larger purchase quantity). The former case follows Taylor’s law, however, the latter case deviates from the law as we confirmed in the stamps category. The basic Poisson behavior, that is, the standard deviation of sales quantities is proportional to the square root of mean value, is realized if the following two conditions are fulfilled: Condition 1; the variance of sales quantities by a purchase is finite. Condition 2; the number fluctuation of customers is smaller than fluctuation of sales quantities.

Future possible research tasks include specifying the factors determining the coefficient of customer quantity variation in the non—Poisson area. Since the boundary value between the Poisson and non—Poisson areas of sales quantities is determined by the coefficient of customer quantity variation, specifying the factors that determine this value is a key issue. This paper analyzed POS data for Kawasaki City, Japan covering summer period (June through October); it will also thus be necessary to make comparisons with scaling laws with wider time and space scales. Such time scales should include scaling laws for winter periods and over one year, and the space scales should include not just a city but an entire prefecture or an even broader region. It will also be necessary to ascertain power indices and changes of boundary values where the power index changes for each category.

As a practical application, consideration of the characteristics of sales fluctuations not only for the sales of a single store but also for the sales totals of multiple stores will help make production quantity management and production delivery timing more accurate throughout the entire retailer chain.

## Supporting Information

S1 Fig CDF and Autocorrelation of some categories. (PDF)

S1 Table The *p* values of the Ljung-Box test. (PDF)

S1 Appendix Derivation of Eq (8). (PDF)

S2 Appendix NBD model. (PDF)

S3 Appendix BBD model. (PDF)
