# Supplementary material for: Property of Fluctuations of Sales Quantities by Product Category in Convenience Stores
Source: PLoS One. 2016 Jun 16;11(6):e0157653. doi: 10.1371/journal.pone.0157653 (PMC4911113; doi:10.1371/journal.pone.0157653)
Supplement: S1 Fig — (PDF) [file pone.0157653.s001.pdf]

S1 Figure: CDF and Autocorrelation of sales time intervals of some categories

S1 Fig shows more examples of CDFs and autocorrelations of sales time intervals of categories, Sandwiches, Breads, Nutrition drinks, and Magazines.

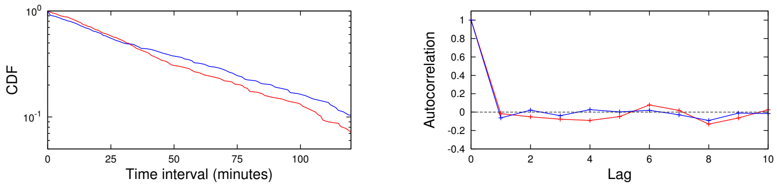

(a) Sandwiches

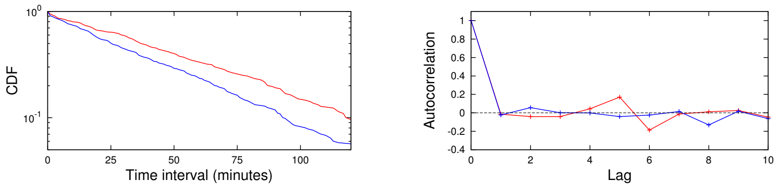

(b) Breads 2

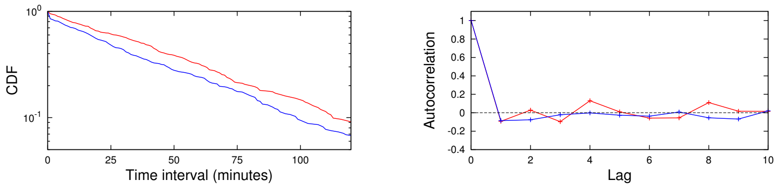

(c) Nutrition drinks

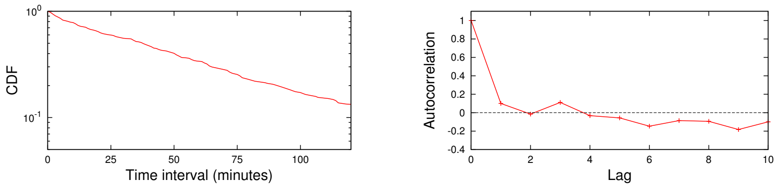

(d) Magazines

S1 Figure **CDFs and autocorrelations of sales times intervals of some categories.** In (a),(b) and (c) red line shows results of daytime, blue shows results of evening. (d) shows result of a weekly magazine on the date of the sale.
