# Supplementary material for: Property of Fluctuations of Sales Quantities by Product Category in Convenience Stores
Source: PLoS One. 2016 Jun 16;11(6):e0157653. doi: 10.1371/journal.pone.0157653 (PMC4911113; doi:10.1371/journal.pone.0157653)
Supplement: S1 Table — (PDF) [file pone.0157653.s002.pdf]

S1 Table: *p* values of Ljung-Box test.

The *p* values of the Ljung-Box test were performed under the null hypothesis that the autocorrelation of purchase intervals would have no correlation. Shown below are the *p* values of the Ljung-Box test.

Table 1: *p* values of Ljung-Box tests

n	1	2	3	4	5	6	7	8	9	10
daytime on weekday	0.673	0.602	0.795	0.718	0.828	0.440	0.459	0.246	0.327	0.386
night on weekday	0.375	0.509	0.398	0.282	0.338	0.361	0.471	0.441	0.404	0.464
daytime on non-weekday	0.054	0.085	0.166	0.264	0.371	0.334	0.438	0.444	0.304	0.320
night on non-weekday	0.306	0.430	0.622	0.649	0.557	0.592	0.517	0.353	0.224	0.244
