# Supplementary material for: Property of Fluctuations of Sales Quantities by Product Category in Convenience Stores
Source: PLoS One. 2016 Jun 16;11(6):e0157653. doi: 10.1371/journal.pone.0157653 (PMC4911113; doi:10.1371/journal.pone.0157653)
Supplement: S1 Appendix — (PDF) [file pone.0157653.s003.pdf]

S1 Appendix: Proof of Eq (8).

To show the proof of Eq (8), first

$$E_S[g(S)] = E_N[E_X[g(S)|N]] \tag{S.1}$$
$$V_S[S] = V_N[E_X[S|N]] + E_N[V_X[S|N]] \tag{S.2}$$

will be proven. Here, $g(S)$ is an arbitrary function of S, and V_X, V_N, and V_S are variances of X, N, and S respectively. The conditional probability density function of S as concerns N is taken to be $f_{S|N}$.

$$\begin{aligned} E_N[E_X[g(S)|N]] &= \sum_n E_X[g(S)|N = n] \cdot f_N(n) \\ &= \sum_n \Big\{ \sum_s g(s) f_{S|N}(s) \Big\} \cdot f_N(n) \\ &= \sum_s g(s) \sum_n f_{S|N}(s) \cdot f_N(n) \\ &= \sum_s g(s) f_s(s) \\ &= E_S[g(S)] \end{aligned}$$

Thus, Eq (S.1) is proven. Using this result,

$$\begin{aligned} V_N[E_X[S|N]] &= E_N[E_X[S|N]^2] - E_N[E_X[S|N]]^2 \\ &= E_N[E_X[S|N]^2] - E_S[S]^2 \end{aligned} \tag{S.3}$$

and

$$\begin{aligned} E_N[V_X[S|N]] &= E_N[E_X[S^2|N] - E_X[S|N]^2] \\ &= E_N[E_X[S^2|N]] - E_N[E_X[S|N]^2] \\ &= E_S[S^2] - E_N[E_X[S|N]^2] \end{aligned} \tag{S.4}$$

From Eq (S.3) and Eq (S.4),

$$\begin{aligned} V_N[E_X[S|N]] + E_N[V_X[S|N]] &= E_S[S^2] - E_S[S]^2 \\ &= V_S[S] \end{aligned}$$

Thus, Eq (S.2) is proven.

Using Eq (S.1), μ_S is calculated.

$$\begin{aligned} \mu_S &= E_S[S] \\ &= E_N[E_X[S|N]] \\ &= E_N[N \cdot E_X[X]] \\ &= E_X[X] \cdot E_N[N] \\ &= \mu_X \cdot \mu_N \end{aligned}$$

Using this result and Eq (S.2), $V_S[S]$ is calculated.

$$\begin{aligned} V_S[S] &= V_N[E_X[S|N]] + E_N[V_X[S|N]] \\ &= V_N[N \cdot E_X[X]] + E_N[N \cdot V_X[X]] \\ &= E_X[X]^2 \cdot V_N[N] + V_X[X] \cdot E_N[N] \\ &= \mathrm{CV}(N)^2 \mu_S^2 + \frac{\sigma_X^2}{\mu_X} \mu_S \end{aligned} \tag{S.5}$$

Thus, the scaling law of the fluctuation of S becomes

$$\sigma_S = \sqrt{\frac{\sigma_X^2}{\mu_X}\mu_S + \mathrm{CV}(N)^2 \mu_S^2} \tag{S.6}$$

and Eq (8) is proven.
