# Supplementary material for: Property of Fluctuations of Sales Quantities by Product Category in Convenience Stores
Source: PLoS One. 2016 Jun 16;11(6):e0157653. doi: 10.1371/journal.pone.0157653 (PMC4911113; doi:10.1371/journal.pone.0157653)
Supplement: S2 Appendix — (PDF) [file pone.0157653.s004.pdf]

S2 Appendix: NBD model.

The NBD model is often used in research on consumer purchasing behavior [26–28]. In this model, the number of items X purchased of a single product by a single customer is assumed to be a Poisson distribution, and, considering that there are differences in the mean purchase values among individuals (i.e., reflecting differences in the characteristics of consumers), the Poisson parameter is assumed to follow a gamma distribution. The term " negative binomial distribution " reflects the fact that the parameter is the result of a mixture of the Poisson distribution as it follows a gamma distribution. A simple explanation of this model is provided below.

The number of items X purchased of a single product by a single customer during a certain time period is said to follow the Poisson distribution of the mean λ. Further, considering the differences in consumer characteristics, the Poisson parameter λ is assumed to follow the gamma distribution $\Gamma(r, \alpha)$. In other words, X and λ follow the distribution functions $f(X|\lambda)$ and $g(\lambda|r, \alpha)$ respectively:

$$f(X|\lambda) = \frac{\lambda^X}{X!}\exp(-\lambda) \tag{S.7}$$

$$g(\lambda|r, \alpha) = \frac{\alpha^r}{\Gamma(r)}\exp(-\alpha\lambda)\lambda^{r-1} \tag{S.8}$$

The unconditional probability density of X, which does not depend on individual persons, becomes the negative binomial distribution $\mathrm{NB}(r, \frac{\alpha}{\alpha+1})$ shown in Eq (S.9):

$$f(X) = \frac{\Gamma(X+r)}{\Gamma(X+1)\Gamma(r)}\Big(\frac{\alpha}{\alpha+1}\Big)^r\Big(\frac{1}{\alpha+1}\Big)^X \tag{S.9}$$

The mean $E_X[X]$ and the variance $V_X[X]$ become, respectively,

$$E_X[X] = \frac{r}{\alpha} \qquad V_X[X] = E_X[X] + \frac{1}{r}E_X[X]^2 \tag{S.10}$$

When the number of items X purchased at a single time is made to follow the negative binomial distribution of Eq (S.9), from Eq (S.10), the scaling law (Eq (8)) becomes

$$\sigma_S = \sqrt{\mu_S\Big(1+\frac{1}{\alpha}\Big) + \mathrm{CV}(N)^2\mu_S^2} \tag{S.11}$$

and Eq (9) and (10) become

$$\sigma_S = \sqrt{1+\frac{1}{\alpha}+\mu_X}\cdot\mu_S^{1/2} \qquad (\mu_S \ll A_m = (1+\alpha^{-1})\mathrm{CV}(N)^{-2}) \tag{S.12}$$

$$\sigma_S \approx \mathrm{CV}(N)\cdot\mu_S \qquad (\mu_S \gg A_m = (1+\alpha^{-1})\mathrm{CV}(N)^{-2}) \tag{S.13}$$

Thus, even when the sales-quantity mean μ_S is the same, when differences in consumer characteristics are considered for X, it has a larger fluctuation σ_S than when it follows the Poisson distribution. When the scale parameter α of the gamma distribution satisfies $\alpha \gg 1$, X has approximately the same results as when it is following the Poisson distribution.
