# Supplementary material for: Property of Fluctuations of Sales Quantities by Product Category in Convenience Stores
Source: PLoS One. 2016 Jun 16;11(6):e0157653. doi: 10.1371/journal.pone.0157653 (PMC4911113; doi:10.1371/journal.pone.0157653)
Supplement: S3 Appendix — (PDF) [file pone.0157653.s005.pdf]

S3 Appendix: BBD model.

Like the NBD model, the BBD model is used in research on consumer purchasing behavior [29–31]. One difference between the two depends on whether an upper limit for item purchases exists. The BBD model uses a binomial distribution whose ratio parameter follows a beta distribution, in which item purchase quantities are limited. Conversely, since an NBD model uses a negative binomial distribution, there is no upper limit to item purchase quantities. A BBD model can be used when one is interested in how many issues of a monthly magazine will be purchased over a one-year period. A simple explanation of this model is provided below.

In a BBD model, the number of items X purchased of a single product by a single customer is expressed as the binomial distribution $\mathrm{Bin}(n, \alpha)$. However, considering differences in consumer characteristics, the ratio parameter α is made to follow a beta distribution $\mathrm{Beta}(p, q)$. In other words, X and α follow the distribution functions $f(X|\alpha)$ and $g(\alpha|p, q)$ respectively:

$$f(X|\alpha) = \binom{n}{X}\alpha^X(1-\alpha)^{n-X}, \qquad X = 0, 1, 2, \cdots, n \tag{S.14}$$

$$g(\alpha|p, q) = \frac{1}{\mathrm{B}(p, q)}\alpha^{p-1}(1-\alpha)^{q-1} \tag{S.15}$$

Here, $\mathrm{B}(p, q)$ is a beta function defined with the following equation:

$$\mathrm{B}(p, q) = \int_0^1 y^{p-1}(1-y)^{q-1}dy$$

The unconditional probability density $f(X)$ of X, which does not depend on individual persons, becomes

$$f(X) = \binom{n}{X}\frac{\mathrm{B}(p + X, q + n - X)}{\mathrm{B}(p, q)}, \qquad X = 0, 1, 2, \cdots, n \tag{S.16}$$

Now, while ignoring cases of sales wherein a single customer purchases two or more of the same item during a single purchase, the parameter n that sets an upper limit in the binomial distribution is taken as 1. Thus, when X is assumed to follow a Bernoulli distribution, Eq (S.16) reverts to the following binomial distribution in $\mathrm{Bin}(1, \frac{p}{p+q})$:

$$f(X) = \Big(\frac{p}{p+q}\Big)^X\Big(\frac{q}{p+q}\Big)^{1-X}, \qquad X = 0, 1 \tag{S.17}$$

Here, when $a \equiv \frac{p}{p+q}$, $b \equiv \frac{q}{p+q}$, the mean $E_X[X]$ and the variance $V_X[X]$ respectively become

$$E_X[X] = a \qquad V_X[X] = ab \tag{S.18}$$

Thus, the scaling law (Eq (8)) of the mean μ_S and the standard deviation σ_S of sales quantities S becomes

$$\sigma_S = \sqrt{b\mu_S + \mathrm{CV}(N)^2\mu_S^2} \tag{S.19}$$

The fact that the property of the Poisson area can be expressed without the use of an approximation is a special characteristic of the BBD model. From Eq (8) and (S.18)

$$\begin{aligned}\sigma_S &= \sqrt{\frac{\sigma_X^2}{\mu_X} + \mu_X} \cdot \mu_S^{1/2} \\ &= \sqrt{b + a} \cdot \mu_S^{1/2} \\ &= \mu_S^{1/2}\end{aligned}$$

Further, when the customer-quantity time series in the Poisson area is assumed to follow a Poisson distribution, the fact that the sales quantity S also follows a Poisson distribution can be proven as follows using a moment-generating function. X is made to follow Eq (S.17), and N follows the Poisson distribution of the mean λ_N. The moment-generating functions of X, N, and S are taken as $M_X(t)$, $M_N(t)$, and $M_S(t)$.

$$
\begin{aligned}
M_S(t) &= E_S[\exp(St)] \\
&= M_N(\log M_X(t)) \\
&= \exp\left\{-\frac{p}{p+q}\lambda_N(1-\exp t)\right\}
\end{aligned}
$$

This is moment-generating function of the Poisson distribution whose mean is $\frac{p}{p+q}\lambda_N$. Thus, it is proven that S follows the Poisson distribution whose mean is $\frac{p}{p+q}\lambda_N$.
